# Orbital nocardiosis

**DOI:** 10.4103/0301-4738.77021

**Published:** 2011

**Authors:** Suryasnata Rath, Savitri Sharma, Geeta K Vemuganti

**Affiliations:** Ophthalmic Plastic and Reconstructive Surgery, Orbit and Ocular Oncology Service, L V Prasad Eye Institute, Bhubaneswar-751 024, India; 1 Ocular Microbiology Service, L V Prasad Eye Institute, Bhubaneswar-751 024; 2 Ophthalmic Pathology Service, L V Prasad Eye Institute, Hyderabad-500 034, India

Dear Editor,

We read with interest the rare case of disseminated orbital actinomycetoma reported by Shanbagh *et al*.[1] The authors have crafted a good report highlighting the clinical features, radiological findings, and management of orbital actinomycetoma caused by *Nocardia brasiliensisbrazilienses / Nocardia asteroides*.[1] We would, however, like to bring to the fore a few issues not addressed by the authors.

The authors’ claim to be the first in reporting orbital invasion of actinomycetoma is misplaced. A quick literature search revealed a report by Patil *et al*., which described a 26-year-old male who was found to have multiple discharging sinuses on the scalp, with orbital and intracranial invasion.[2] Interestingly the presentation, clinical features, imaging, and histopathological details of the two patients have an uncanny resemblance and the ‘patients’ and authors are from the same hospital.[1,2]

Figure 3 in the article is wrongly labelled as computed tomography (CT) scan of orbit while it shows pictures of magnetic resonance imaging (MRI). The authors have described the osteolytic changes seen in longstanding undiagnosed cases of actinomycosis.[1] However, in the interest of the readers, this needs further emphasis and documentation. Patil *et al*. have described the characteristic moth-eaten appearance of the frontal bone along with orbital and intracranial invasion, in the case reported by them.[2]

It may be of interest to note that orbital actinomycosis has been also reported by Pagliani and Sullivan *et al*.[3,4] Pagliani *et al*. described a case of orbital actinomycosis in a patient with a history of carcinomas of the breast and kidney.[3] A CT scan of brain and orbit in this patient showed multiple orbital lesions and widening of the ipsilateral cavernous sinus.[3] Sullivan *et al*. described a case of orbital actinomycosis with a small ill-defined lesion in the posterolateral orbit.[4] The significant difference was that the periocular skin was uninvolved in both the above-mentioned cases.

## References

[1] Shanbhag NU, Karandikar S, Deshmukkh PA (2010). Disseminated orbital actinomycetoma: A case report. Indian J Ophthalmol.

[2] Patil SP, Gautam MM, Sodha AA, Khan KJ (2009). Primary cutaneous nocardiosis with craniocerebral extension: a case report. Dermatol Online J.

[3] Pagliani L, Campi L, Cavallini GM (2006). Orbital actinomycosis associated with painful ophthalmoplegia. Actinomycosis of the orbit. Ophthalmologica.

[4] Sullivan TJ, Aylward GW, Wright JE (1992). Actinomycosis of the orbit. Br J Ophthalmol.

